# Dose the increasing burden of social endowment affect sustainable development of economy?

**DOI:** 10.1371/journal.pone.0296512

**Published:** 2024-01-02

**Authors:** Zhiyang Yu, Jin Chen, Runfa Yu

**Affiliations:** 1 School of Banking and Finance, University of International Business and Economics, Beijing, China; 2 College of Foreign Languages, Xinjiang University, Urumqi, Xinjiang, China; Federal University of ABC, BRAZIL

## Abstract

The rapid increase in the number of older people under the background of population aging has gradually changed the disease spectrum of society, making aging diseases more prevalent, and increasing the demand for health care services, medical and health services, and health insurance among older people, ultimately leading to increasing household and social spending on old age. This study is conducted to assess the impact of those spending burden on the sustainable development of economy and find out some practical and effective solutions. This paper constructs a theoretical model to illustrate the relationship between the old-age dependency ratio and the marginal product of capital (MPK), and then establishes a two-way fixed effect model based on transnational panel data of 81 countries from 1981 to 2017 to verify this relationship empirically. This paper finds that, after controlling a series of variables, an increased burden of old-age dependency leads to a decline in the MPK, a key macroeconomic variable and also a sustainable development criteria, but in which health care, health security systems, and technological innovation play a key and moderating role. The conclusion is also valid after tackling the problem of endogeneity with different methods, like two-stage least squares (TSLS) and the generalized methods of moments (GMM). Overall, before population aging, countries that are old-but-not-rich should encourage more supply-side investments in public health system or technological innovation, and adjust retirement system, or gradually encourage childbearing to strive for time and space for later sustainable development of public health system and economy.

## Introduction

In the past 20 years, the conditions of health care, the level of older people services and health security have been improved, and the proportion of people with access to healthy drinking water and health services has increased by 2.6% and 25.4% respectively. The global life expectancy has increased from 67.5 years to 72.8 years, which has greatly reduced human mortality, while the global fertility rate has decreased from 3.3 to 2.4 and birth rate from 25.9 to 17.3. This has led to an increasing proportion of older people. For example, since 2000, the global old-age dependency ratio has increased from 10.9 to 14.6, and health expenditures have risen sharply from 598.5 to 1,467 international dollars, an increase of nearly 1.5 times, while per capita income has increased only less than 1.2 times over the same period. All the data above are from the WDI database of the World Bank. It can be seen that aging is having a significant impact on the economy, and some scholars have also found that aging is an important reason for secular stagnation [1–4]. Therefore, in order to achieve sustainable economic development, this topic deserves continuous attention from scholars around the world. This paper reviews literature related to the research objective of this study and categorizes it into the following four categories.

From the perspective of indicators describing the degree of aging, existing literature has proposed many indicators, both traditional and new. For example, the traditional old-age dependency ratio can be used to describe both the burden of social endowment and the degree of aging, and the United Nations’ indicator of the proportion of elderly people aged 65 (or 60) and above to the total population is also the same However, the age in these indicators is based on the "retrospective age", which means how long one has lived. Other scholars have proposed indicators based on "prospective age", such as the prospective old-age dependency ratio, which is based on life expectancy [5–7]. However, these measures do not consider the differences in health and functional status between people of the same chronological age. Many of those aged 60 or 65 years old and over could still be healthy and working, which reduces the social burden instead. Therefore, in order to better judge the actual situation of aging, other dimensions need to be added to the judgment criteria. Currently, there are four dimensions as follows: The first is to consider the physical health status. For example, one indicator of physical health to measure older people is HALE, meaning the average time a person can survive in good health for a given condition of mortality and morbidity [8]. This indicator has been widely used by the World Health Organization, the United Nations and other international agencies. The second is physical functioning status, which contains three specific sub-dimensions: cognitive function, sensory function and physical function. Among them, cognitive function refers to the mental functions of attention, thinking, understanding, learning, memory, problem solving and decision making [9]; sensory function mainly includes vision and hearing, which are also closely related to cognitive function [10]; physical function is measured by blood pressure, blood oxygen saturation, heart rate, lung capacity, gait speed, standing balance, grip strength, chair stand test and reflex speed were assessed [11]. The third is biomarkers. People show some characteristics during aging, such as height, weight, body mass index, muscle strength, skin wrinkles or facial features and hair color [12, 13]. The fourth is personal subjective judgment, which mainly asks participants about their subjective judgment of their physical state at the moment [14]. Based on the analysis above, the traditional indicators do have some shortcomings, but the new ones has high requirements for data, which is not enough to carry out systematic transnational analysis. Therefore, this paper still uses the traditional old-age dependency ratio and the proportion of elderly population indicators to measure the burden of social endowment brought by aging.

From the perspective of the possible economic impacts of aging, some scholars have focused on studying the relationship between aging and technological progress or labor productivity, but have not formed a consensus. For example, in terms of the relationship between aging and economic growth, Kremer [15] finds through theoretical and empirical tests that a reduction in the total population reduces the rate of technological progress, which in turn affects the growth rate of the total population. Feyrer [16] empirically finds that population aging leads to slower growth in total factor productivity, which in turn leads to slower growth in output per capita. However, Izmirlioglu [17] finds that the aging population age structure does not necessarily impede technological progress, but rather leads to an increasing share of R&D. Maestas et al. [18] empirically test the negative impact of the rate of population aging on economic growth based on the cross-state data in the United States, and find that population aging negatively affects the economy mainly through its impact on labor productivity. However, Acemoglu & Restrepo [19] believe that because of the similar level of technology application between different states, it is difficult to identify the positive role of technology investment related to aging. In view of this, they use the transnational panel data to re-examine this relationship and find a significantly positive effect of aging on economic growth.

Some scholars have also conducted research from the perspective of the relationship between aging and savings or aging and investment, but the conclusions are not very consistent. Musgrove [20] find that the relationship between household saving rate and household size is not unique, and there are large transnational differences. Leff [21] studies the relationship between the dependency ratio and savings rate using cross sectional data and find that the two are inversely proportional, but Ram [22] refuted this view after using cross sectional data and the same method. Kelley [23] believed that the impact of dependency ratio and population growth on the savings rate would vary with countries, and the reverse relationship between the two was not established. Li et al. [24] empirically found that life expectancy has a positive impact on savings and investment, while population structure is the opposite.

In addition to the above three aspects, there are also many scholars who have conducted studies from the perspective of the relationship between demographic factors and interest rates [25–29]. And most scholars believe that aging is now depressing and will continue to depress the real interest rate [25, 28, 29].

After reviewing the literature, this paper finds that there is currently no consensus on the impact of population aging on economic or social development. It still needs to be continuously validated from new perspectives and using new data, in order to better understand the economic impact and mechanism of aging and provide valuable policy recommendations for better addressing the negative effects of aging in the future.

The main research content of this paper is to analyze whether the increase in the burden of social endowment has an impact on the marginal product of capital under the background of aging by constructing theoretical and empirical models, and to test the robustness of the conclusion by using the instrumental variable method to handle the endogeneity problem. Finally, the sample data are cross grouped to test whether there is heterogeneity in the impact of the burden of social endowment on marginal product of capital, which helps to infer the impact of the burden of social endowment on sustainable economic development and its characteristics. The research also has certain theoretical and practical value. Firstly, regarding the topic of the relationship between aging and economic development, this paper finds that there is relatively little theoretical and empirical research on MPK. However, MPK is an important indicator for measuring long-term sustainable economic development and a key macroeconomic variable. Therefore, this paper preliminarily studies the impact of the burden of social endowment under the background of aging on MPK from both theoretical and empirical perspectives, filling the gaps in this field and helping to comprehensively understand the economic impact of aging. Secondly, the conclusions of this paper help to extend the analysis of other important economic issues, such as the real interest rate decided by marginal product of capital theoretically. Therefore, the conclusions of this paper can extend the analysis of the impact of aging on the real interest rate. In addition, according to Tobin Q theory, the impact of aging on the real interest rate and marginal product of capital can be analyzed simultaneously to further analyze the impact of aging on investment. Investment is the core tool of macroeconomic regulation policies, so this study has a relatively broad research significance.

The rest of this paper proceeds as follows: Section 2 is the “Materials and Methods”, including study design, study tool and data collection. In this section, this paper constructs a theoretical model to illustrate the relationship between the old-age dependency ratio and MPK, establishes a two-way fixed effect model based on the theoretical model, and makes use of transnational panel data of 81 countries from 1981 to 2017 to verify that relationship empirically. Section 3 is “Results”, mainly displaying the empirical results and analysis including baseline analysis, endogeneity analysis and heterogeneity analysis. Section 4 is the conclusions and recommendations. This section summarizes the main research conclusions, proposes relevant policy recommendations, and elaborates on the research shortcomings and future research directions.

## Materials and methods

### Study design and study tool

By reviewing relevant literature, this paper finds that existing theories cannot directly explain the relationship between the burden of social endowment and marginal product of capital, lacking direct theoretical basis. Therefore, before conducting empirical research, this paper attempts to directly explore the relationship between them from a theoretical perspective. In the modeling process, this paper uses the old-age dependency ratio to measure the size of social endowment burden. Based on the Cobb Douglas production function with constant returns to scale, a single sector model is constructed to derive the relationship between the burden of social endowment and marginal product of capital. The conclusions of the theoretical model not only reveals the relationship, but also provides a theoretical basis for constructing empirical models and conducting heterogeneity analysis in the following text.

Assuming that the production function is labor-augmented Y = K ^α^(AL) ^1-α^, total population = N, working population = L, older people population = O, children’s population = C, and N = L+O+C. Making L/N = l, O/L = a, C/L = b, then l represents the proportion of the working age population, a represents the old-age dependency ratio, and b represents the children dependency ratio.

After dividing N on both sides of the equation N = L+O+C, it can be concluded that:

$$\frac{L}{N}=1-\frac{O}{N}-\frac{C}{N}=1-\frac{O}{L}\times \frac{L}{N}-\frac{C}{L}\times \frac{L}{N}=1-\frac{L}{N}(\frac{O}{L}+\frac{C}{L})$$ (1)

Substituting L/N = l, O/L = a, C/L = b into Eq (1) and organizing it can obtain:

L=[1−(a+b]×l)×N (2)

By taking the partial derivative of K in the production function and substituting Eq (2) into it, we can obtain:

$$MPK=\alpha K^{\alpha -1}A^{1-\alpha }{\left\{[1-(a+b)\times l]\times N\right\}}^{1-\alpha }$$ (3)

Derivation and simplification of a in the Eq (3) yields:

$$\frac{dMPK}{da}=-\{\frac{l\times \alpha (1-\alpha ){\left(\frac{K}{AN}\right)}^{\alpha -1}}{{\left[1-(a+b)\times l\right]}^{\alpha }}\}$$ (4)

As 0 < a + b ≤ 1, 0 < l < 1, 0 < α < 1, so the denominator in Eq (4) is positive and the numerator items are also positive, so the overall is negative, which indicates that when the old-age dependency ratio increases without changing other conditions, the marginal product of capital will decline. This paper believes that the old-age dependency ratio can affect the marginal product of capital in equilibrium through three potential mechanisms. Firstly, in an aging social environment, the increase in life expectancy and the burden of social endowment improve people’s propensity to save. Savings gradually increase, consumption continue to decline, and total demand continue to decrease, ultimately leading to a decrease in marginal product of capital in equilibrium. Mason [30] believes that the consumption level of elderly people is generally low. Except for a few high-income countries where the consumption level has increased due to large government subsidies, the consumption level of elderly people in other countries is basically decreasing. Secondly, the labor force itself is a factor input. When the degree of population aging increases, the proportion of the labor force in the total population decreases, leading to a decline in potential economic growth rate and a continuous decrease in the marginal product of capital in equilibrium. Thirdly, in an aging society, the tendency to save increases, the working population decreases, and then capital continues to deepen. Under the law of diminishing returns, the marginal product of capital will continue to decline.

Therefore, this paper proposes the following first hypothesis: Other things being equal, the marginal product of capital will keep decreasing as the burden of social endowment keeps increasing in the aging process.

Continuing the partial derivative of A in Eq (4) and simplifying it yields:

$$\frac{\partial^{2}MPK}{\partial a\partial A}=-(1-\alpha )\{\frac{l\times \alpha (1-\alpha ){\left(\frac{K}{N}\right)}^{\alpha -1}}{{\left[1-(a+b)\times l\right]}^{\alpha }}\}\frac{1}{A^{\alpha }}$$ (5)

A represents technological innovation, as well as public health systems and management systems that are conducive to the survival and development of the elderly, especially in this study. The richer and more developed a country is, the higher the value of A. Equation (5) is negative overall. Therefore, if A is higher, the negative impact of the burden of social endowment caused by population aging on the marginal product of capital is smaller. This paper believes that there are two reasons behind this effect: Firstly, developed countries have more comprehensive healthcare systems, even including easily overlooked oral health care, as well as more advanced pension security and drug production systems, emphasizing human life cycle management. Janto et al. [31] believe that the dental health of the elderly is often neglected because there are many common diseases in this population, such as cardiovascular diseases. However, dental health affects the overall health and quality of life by affecting both the health and psychological status of individuals. In addition, developed countries have well-established medical insurance and pension insurance markets, which to some extent alleviate the burden of social endowment. Moreover, timely introduction and continuous improvement of the deferred retirement policy have made elderly people healthier, their income more stable, and their time out of the labor market has been greatly delayed, reducing the negative impact of aging on the marginal product of capital. For example, in the last decades, governments have tried to increase the labor force participation by raising retirement age [32], and Carta F et al. [33] who quantifies the effect of a policy-induced sharp increase in retirement ages on input mix and economic outcomes of firms using Italian matched worker-firm data find that the rising institutional retirement ages can help firms to retain valuable older employees in Italy. Secondly, developed countries have strong technological innovation capabilities, master core technologies, and invest heavily in scientific research, which promotes the application of labor substitution technology and compensates for the negative impact of insufficient labor force on marginal product of capital. Therefore, even though developed countries have severe aging problem, their advanced public health systems, management systems, and technological advancements have greatly alleviated the negative impact of aging.

Therefore, this paper proposes the second hypothesis: Countries that are old-and-rich are less affected than countries that are old-but-not-rich, and there is heterogeneity.

It should be emphasized that in order to reveal the theoretical relationship between the burden of social endowment and marginal product of capital, this paper attempts to construct a single sector model, which is an extreme simplification of the real world. Therefore, scholars interested in this topic can also try to construct a multi sector model that is more consistent with reality in the future.

The benchmark model in this paper uses a two-way fixed effects model, referring to Honda and Miyamoto [34]. Based on the MPK equation derived from the above theoretical model, the specific form of the empirical model is set as follows:

$$MPK_{it}=\alpha +\beta Burdn_{it}+\gamma Control_{it}+\lambda_{t}+\mu_{i}+\varepsilon_{it}$$ (6)

In Eq (6), i and t represent country and time, λ_t_ and μ_i_ represent time and individual fixed effects, the core independent variable is the burden of social endowment, Burdn_it_, expressed as the old age dependency ratio. Control_it_ represents control variables, α, β, γ, and ε_it_ respectively represent the intercept term, the coefficients of the core explanatory variables, the coefficients of the control variables, and the random disturbance term. The dependent variable is MPK, which is calculated by deriving the Cobb-Douglas production function with constant returns to scale. For the selection of control variables, in addition to those involved in the theoretical model MPK equation, based on other theories and practice in economics, this paper adds capital structure, human capital, foreign dependence, government consumption, urbanization, and banking crisis, as detailed in Table 1. In the theoretical model, l is influenced by the old-age and juvenile dependency ratios, so l is no longer added to the control variables to avoid increasing multicollinearity. Specifically, since the efficiency of private capital is higher than that of public capital, the capital structure can express the impact of capital operation efficiency on MPK; In addition to technological progress in the labor-enhanced Cobb-Douglas production function, other factors, especially human capital, will also affect GDP per capita and even MPK; Other structural factors that cannot be reflected in the single-sector growth model, including trade dependence reflecting the degree of openness, government consumption reflecting the degree of government intervention in the economy and urbanization rate reflecting the urbanization process, may have an impact on MPK.

**Table 1:** Variable description.

Variable name	Proxy variables	Variable type	Specific instructions	Source
**Marginal product of capital**	MPK	Dependent variable	Using the formula equation	PWT
			Replace with the internal real rate of return (Irr)	
**The burden of social endowment**	Burdn	Independent variable	Using the old-age dependency ratio to express	WDI
**Capital stock**	K.stock	Control variables	Using the logarithmic representation of capital stock under the 2011 PPP method to express	IMF
**Capital structure**	K.struc		Using the ratio of private sector to government sector capital stock to express	
**GDP per capita**	GDP.percap		Calculated and logged using GDP under the 2011 PPP method	
**Trade dependence**	Trade.dep	Control variables	Using the sum of imports and exports as a percentage of GDP to express	WDI
**Government consumption**	Gov.cons		Using government final consumption as a percentage of GDP to express	
**Urbanization**	Urban		Using urban population as a percentage of total population to express	
**Banking crisis**	Bank.cris		Using 1 and 0 to express	
**Population growth**	Pop		Using the annual population growth rate to express	
**Child support ratio**	Csup.ratio		Using population aged 0–14 years/working population aged 15–64 years to express	
**Capital share**	K.share	Control variables	Using "1—labor share" to express	PWT
**Human capital**	HCI		Using the Human Capital Index to express	

All the sample data were analyzed statistically using Stata version 15.1. *p-value* < 0.1 was considered statistically significant, and the lower *p-value*, the more significant result.

### Data collection

The sample data in this paper covers 81 countries or regions worldwide from 1981 to 2017, sourced from the World Bank’s WDI database, IMF databases, and The Penn World Table (PWT) database. The data filter criteria are as follows: Firstly, this paper removes countries or regions where observation values have been missing for more than 3 consecutive years. Second, other missing data is supplemented by taking the average of neighboring years. The government consumption data of Argentina from 1981 to 1986 was replaced by 1979 data, mainly considering that Argentina is an important economy and there are no missing other data, so it has been retained. Therefore, the sample in this paper is a balanced panel. This paper performs logarithmic processing on capital stock and per capita GDP data, and the data description is detailed.in Table 1. During the sample period, the capital stock data of the public-private partnership component is missing for more years and accounts for a smaller portion of the total capital stock, so this component is not included in the capital stock.

## Results

### Baseline analysis

Because the variables in the benchmark model are commonly used and cannot be ignored in macroeconomic research, and most of them come from the theoretical derivation mentioned earlier, they are likely to have long-term stable equilibrium relationships in theory. In addition, this paper used Kao’s [35] method for cointegration testing and found to reject the original hypothesis of "no cointegration". Therefore, there is a long-term stable equilibrium relationship between the variables in the benchmark model, and the regression results at this time are more accurate. In Table 2, columns (1) and (3) show the results with some control variables, while columns (2) and (4) show the results with all control variables. When all control variables are included, the maximum VIF value and the overall average VIF value in the benchmark model are far less than 10, indicating that there is no serious multicollinearity problem in the benchmark model setting, and the regression results have a certain degree of reliability. The results of the benchmark regressions show that the direction of the impact of the burden of social endowment (Burdn) on MPK is consistent with the theoretical model in study design, and there is a significant negative correlation at 1% or 5% level between the two. Replacing the dependent variable with the percentage of population aged 65 and older or replacing MPK with the internal real rate of return (Irr), the conclusion remains the same. Comparing column (2) with column (4), the absolute value of the Burdn coefficient is larger and the significance is increased with better estimation when both individual and time fixed effects are considered. Referring to the results in column (4), each 1% increase in the Burdn will lead to a decrease in MPK by about 0.582%. This holds at the 1% significance level and hypothesis one is tested. The coefficients on the control variables capital share, capital stock, and per capita income are also significant at the 1% level, and the coefficient on the child support ratio is negative at least at the 10% level of significance.

**Table 2:** Baseline regression results.

	(1)	(2)	(3)	(4)
**Burdn**	-0.403** (0.159)	-0.360** (0.152)	-0.636*** (0.182)	-0.582*** (0.174)
**K.share**	0.626*** (0.0723)	0.617*** (0.0742)	0.577*** (0.0674)	0.569*** (0.070)
**K.struc**	-0.965 (0.627)	-0.918 (0.625)	-0.786 (0.511)	-0.811 (0.521)
**K.stock**	-15.84*** (1.291)	-16.05*** (1.421)	-18.15*** (1.249)	-18.22*** (1.382)
**GDP.percap**	16.28*** (1.910)	16.22*** (1.968)	17.10*** (1.941)	17.16*** (1.950)
**HCI**	6.534*** (1.556)	5.827*** (1.757)	0.489 (2.273)	0.253 (2.521)
**Csup.ratio**	-0.110*** (0.039)	-0.102** (0.045)	-0.084** (0.037)	-0.071* (0.041)
**Pop**		0.469** (0.217)		0.256 (0.176)
**Trade.dep**		-0.004 (0.007)		-0.006 (0.008)
**Gov.cons**		-0.082 (0.082)		-0.100 (0.066)
**Urban**		0.097 (0.075)		0.074 (0.068)
**Bank.cris**		-0.721** (0.275)		-0.573** (0.245)
**Constant term**	257.3*** (21.32)	259.6*** (22.91)	324.4*** (21.24)	322.9*** (23.94)
**Time fixed effects**	No	No	Yes	Yes
**Individual fixed effects**	Yes	Yes	Yes	Yes
**R** ^**2**^	0.646	0.660	0.705	0.714
**N**	2997			
**Number of countries or regions**	81			

Note

"***", "**" and "*" indicate significance levels at 1%, 5% and 10% respectively.

### Endogeneity analysis

According to controlled experiment in medical experiments and econometrics, the endogeneity of the benchmark regression model mainly comes from omitted control variables and two-way causality, which lowers the reliability of experiment results. Therefore, firstly, this paper selects control variables from both social structure and cultural level to solve the problem of omitted control variables as much as possible. Secondly, the two-way causality problem in the benchmark regression model is mainly reflected in the following two aspects: on the one hand, the burden of social endowment affects MPK, and on the other hand, MPK may have a feedback effect on the burden of social endowment by increasing investment or income, etc. The instrumental variable method plays a very important role in economic research, as its main role is to solve endogeneity problems and obtain more accurate research results. Therefore, if the instrumental variable method is not used, the conclusions drawn may differ significantly from the actual situation, which will have a serious impact on policy formulation and implementation. Therefore, in the following text, the instrumental variable method will be used to address the endogeneity problem caused by two-way causality.

For the scientific and rigorous nature of the experiment, four instrumental variables are selected and then divided into two groups to prove each other, where the first group is the main group and the second group is used for auxiliary analysis. The two groups are the first-order lag term for fertility rate and mortality rate and the first-order lag term for sex ratio and net birth rate (the difference between birth rate and mortality rate) respectively, and the regression results and their robustness under two different sets of instrumental variables are tested separately in the following.

Effectiveness analysis of instrumental variables: Firstly, all four instrumental variables are closely related to the burden of social endowment under the background of aging, but there are differences in the perspectives, which can meet the correlation requirements of instrumental variables. Specifically, on the one hand, the imbalance in the sex ratio with a lag of one period will affect future marriage rate, further affecting fertility rate, and ultimately affecting the number of newborns. The working population will not be effectively replenished, and the continuous decline in the net birth rate with a lag of one period will also have the same result in the future. On the other hand, as the first-order lag term of mortality decreases, the phenomenon of longevity will become increasingly common, and the elderly population will gradually increase in the future. The labor force cannot be supplemented while the number of elderly people continues to increase. As a result, the population structure is gradually aging, and the overall burden of social endowment is constantly increasing. Therefore, these four instrumental variables meet the correlation requirement. Secondly, the first-order lag term of these four instrumental variables belongs to the pre-determined variable and their value is fixed, so it will not be related to the current disturbance term in the model. So these four instrumental variables also meet the exogenous requirements. Through the above analysis, it can be concluded that the first-order lag terms of fertility rate, mortality rate, sex ratio, and net birth rate are all effective instrumental variables that can be used to effectively address endogeneity issues in the benchmark model.

Validity test of instrumental variables: The first aspect is the correlation test of instrumental variables. According to the rule of thumb, if the first stage F-statistic of the two-stage least squares method is greater than 10, the original assumption of "the existence of weak instrumental variables" will be rejected. In Table 3, the F-statistic in columns (1)—(4) is much greater than 10, indicating that the instrumental variable meets the correlation requirement. The second aspect is the exogenous test of instrumental variables. In Table 3, the results of the over identification test are as follows: in the first regression result, the Sargan statistic is equal to 0.087, the *p*-value is 0.769, the Hansen-J statistic is equal to 0.066, and the *p*-value is 0.797. In the second regression result, Sargan statistic equals 0.116, *p*-value equals 0.733, Hansen-J statistic equals 0.046, *p*-value equals 0.831. Both results accept the original assumption that all instrumental variables are exogenous, indicating that all instrumental variables meet the exogenous requirement. Based on all the test results, this paper believes that all instrumental variables meet both the correlation and the exogenous requirement. Therefore, all instrumental variables are effective instrumental variables that can be used for the estimation of the instrumental variable method, ensuring the unbiased and consistent estimation of the core explanatory variable social pension burden.

**Table 3:** Instrumental variables method regression results.

	(1)	(2)	(3)	(4)
	tsls1	gmm1	tsls2	gmm2
**Burdn**	-1.443*** (0.320)	-1.443*** (0.077)	-0.736** (0.307)	-0.736*** (0.096)
**Control variables**	Yes	Yes	Yes	Yes
**Two-way fixed effect**	Yes	Yes	Yes	Yes
**F statistic**	268.499	131.794	117.010	94.911
**LM statistic**	457.999 (0.000)	257.841 (0.000)	219.610 (0.000)	129.292 (0.000)
**Sargan or Hansen-J**	0.087 (0.769)	0.066 (0.797)	0.116 (0.733)	0.046 (0.831)
**DWH**	214.778 (0.000)		2.728 (0.099)	
**R** ^**2**^	0.613		0.715	
**N**	2916			
**Number of countries or regions**	81			

Note

"***", "**" and "*" indicate significance levels at 1%, 5% and 10% respectively; data in parentheses at instrumental variable tests are *p-value*s, others are robust standard errors: “F statistic” is “Cragg-Donald Wald F statistic” or “Kleibergen-Paap rk Wald F statistic”, “LM statistic” is “Anderson canon.corr.LM statistic” or “Kleibergen-Paap rk LM statistic”, “DWH” is the statistical quantity of Durbin-Wu-Hausman.

In addition, to verify whether the model has endogeneity issues, this paper uses the more robust Dubin Wu Hausman method under heteroscedasticity to test the endogeneity of the model. In Table 3, the *p*-value of the Durbin Wu Hausman statistic for the first group of models is 0.000, rejecting the original hypothesis that "all explanatory variables are exogenous" at a 1% significance level, while the *p*-value of the second group is 0.099, rejecting the original hypothesis at a 10% significance level. In summary, both models have endogeneity issues and must be addressed using the instrumental variable method.

In Table 3, this paper estimates the models using two-stage least squares (TSLS) and the generalized methods of moments (GMM), which is more efficient in estimating under heteroskedasticity or autocorrelation conditions. In Table 3, column (1)-(2) show the regression results for the first set of instrumental variables and (3)-(4) show the results for the other set of instrumental variables. The empirical results of the GMM remain consistent with theory, the negative effect of the Burdn on MPK still holds significantly at the 1% level, and the errors in the estimators are significantly reduced, improving the efficiency of the estimation.

### Heterogeneity analysis

This section does not adopt the traditional single standard grouping method, such as grouping based on geographical location, social system, economic development level, etc., but instead adopts a new cross grouping method. The main reasons are as follows: Firstly, compared with traditional methods, the conclusions drawn after cross grouping have stronger explanatory power for the actual situation, and the policy insights obtained are more targeted and have more benefits. The fundamental reason behind this is that after cross grouping, the differences between samples are smaller, there are more commonalities, and the bias of the results is also smaller. For example, if we only group countries based on whether they are aging countries, we will conclude that countries with more severe aging have a greater negative impact on their marginal product of capital. However, after referring to real data from developed countries such as the United States, Britain, Germany, and France, we will find that this is not the case because we overlook the differences in economic development between countries. Secondly, this paper theoretically derives Formula (5) and proposes the corresponding hypothesis. To verify this hypothesis, it is necessary to add another criterion for whether it is a developed country to the grouping criteria for whether it is an aging country, in order to test the differences in the impact of aging between developed countries and other countries.

According to the United Nations’ classification criteria, this paper uses whether the percentage of the population aged 65 and above in the total population of each country reaches or exceeds 7% in 2017 as the basis for determining whether a country has entered the aging stage. If the percentage of one country reaches or exceeds 7%, it is considered an “old country”. Meanwhile, according to the World Bank’s criteria, this paper uses whether the GNI per capita in 2017 exceeds $12055 to determine whether a country is a “rich country”, which is defined by the World Bank as a high-income country. Then the full sample was cross-grouped according to the degree of aging and income level, and the following three categories of sub-samples were identified: old-but-not-rich (13 countries), old-and-rich (33 countries) and not-old-not-rich (33 countries). There are 35 rich countries among the 81 countries in this paper, of which two countries (Saudi Arabia and Bahrain) do not meet the 7% aging criterion, so there are 33 countries after deletion.

As is shown in Table 4, columns (1), (4), and (7) are ordinary least squares estimation results without adding any control variables. Columns (2), (5), and (8) add only some control variables and have the same control variable settings as columns (1) and (3) in Table 2, while columns (3), (6), and (9) include all control variables. Taking columns (3), (6), and (9) as reference, the most significant effect of the increased burden of social endowment on the marginal output of capital under the background of aging is on countries that are old-but-not-rich, followed by countries that are old-and-rich, and countries that are not-old-not-rich, corresponding to 1%, 5%, and 10%, respectively. The absolute value of the coefficient of countries that are old-but-not-rich, 2.565, is much larger than other groups, with significant heterogeneous results. This difference is consistent with the theoretical analysis in study design, which validates hypothesis two.

**Table 4:** Heterogeneity analysis.

	old-but-not-rich			old-and-rich			not-old-not-rich		
	(1) ols1	(2) ols2	(3) ols3	(4) ols1	(5) ols2	(6) ols3	(7) ols1	(8) ols2	(9) ols3
**Burdn**	-3.819**	-2.633***	-2.565***	-0.357**	-0.265***	-0.202**	-0.221	-0.784**	-0.676*
	(1.483)	(0.703)	(0.612)	(0.143)	(0.089)	(0.078)	(0.561)	(0.339)	(0.360)
**Control variables**	No	Part	Yes	No	Part	Yes	No	Part	Yes
**Two-way fixed effect**	Yes	Yes	Yes	Yes	Yes	Yes	Yes	Yes	Yes
***R*** ^**2**^	0.484	0.852	0.871	0.176	0.730	0.781	0.056	0.792	0.804
***N***	481			1221			1221		
**Number of countries or regions**	13			33			33		

## Discussion

The reality is also same as the results in Tables 2–4 On the one hand, higher capital shares and per capita income in high-income countries have enough resources to invest in older people to work longer, which in turn reduces the burden of social endowment and mitigates the impact on MPK. For example, per capita health security expenditures in high-income countries reached 6,242.5 international dollars in 2019, which is about 6, 22 and 58 times higher than in upper-middle, lower-middle and low-income countries during the same period. Moreover, the number of health care workers and beds per 1,000 people in high-income countries were 11.4 and 5.3, compared with only 2.5 and 2.3 in other income countries, which is a significant gap.

On the other hand, developed nations invest more in health care, pension security, research and innovation, with abundant human resources and high per capita income. So the burden of elderly care on households has a smaller impact on MPK. For example, government spending on health security and R&D as a share of GDP is as high as 7.7% and 2.7% in high-income countries in 2019, and only 2.8% and 1.6% in other countries. What’s more, the gap of the human capital index between high-income countries and others is significant, for example, Singapore is 1.35 times higher than China. In Table 4, the negative effect of old-age dependency ratio on marginal product of capital is less significant for the countries that are not-old-not-rich, mainly because these countries have younger demographics, lower industrialization levels and less old-age burden.

## Conclusions

In this paper, under the background of aging, we found a rule from theoretical and experiment perspectives that the increase of the burden of social endowment leads to the decrease of MPK, a key and sustainable development criteria of economy. The conclusion of this paper supports the view that aging will have a negative impact on economic development, not the contrary. It also supports the view that aging will continue to reduce the real interest rate, because according to the neoclassical growth model, the marginal product of capital determines the real interest rate, so when the burden of social endowment increases and the marginal product of capital decreases, the real interest rate will also decrease. In addition, the reasonable prediction based on the research conclusion of this paper can infer that the investment under the background of aging may face the possibility of decline, especially when the marginal product of capital falls faster than the real interest rate and the real interest rate has a lower bound of zero interest rate, Tobin q = MPK/r < 1, the investment will decline.

We found that not the more severely aging countries are, the more they are affected. However, countries that are old-but-not-rich are the most affected. For countries that are old-but-not-rich, they should pay attention to investments in public health care, old-age security and technology innovation before population aging, or pay more attention to system adjustment like delayed retirement policies for older people to delay their exit from the job market, or they should gradually abolish family planning and encourage incentives for childbirth to maintain the size of young and strong labor force, which will boost the sustainable development of society.

There are also some shortcomings in this study. Firstly, this paper attempts to analyze the impact of aging from a new perspective of the marginal product of capital, but this paper only uses simpler calculation method to calculate the transnational data of MPK. With the emergence of new calculation methods and data, more accurate data of MPK should be used for research in the future. Secondly, the indicators used in this paper to measure the level of aging are more traditional, and the new indicators can more accurately reflect the size of a country’s burden of social endowment. Finally, this paper did not conduct research on the transmission mechanism of the impact of the burden of social endowment on marginal product of capital, which is crucial for understanding and responding to the economic impact of aging and is worth further research. Since the marginal product of capital has a significant impact on investment, and investment is an important tool for macroeconomic adjustment and control policies, the impact of aging can be analyzed from the perspective of the specific impact and mechanism of aging on investment in the future.

## Supporting information

S1 Data 1 Data for the baseline and IV regression. 2 Data for the heterogeneity analysis. 3 Notes. (ZIP)
